# Supplementary figures and images for: In silico agent-based modeling approach to characterize multiple in vitro tuberculosis infection models
Source: PLoS One. 2024 Mar 22;19(3):e0299107. doi: 10.1371/journal.pone.0299107 (PMC10959380; doi:10.1371/journal.pone.0299107)

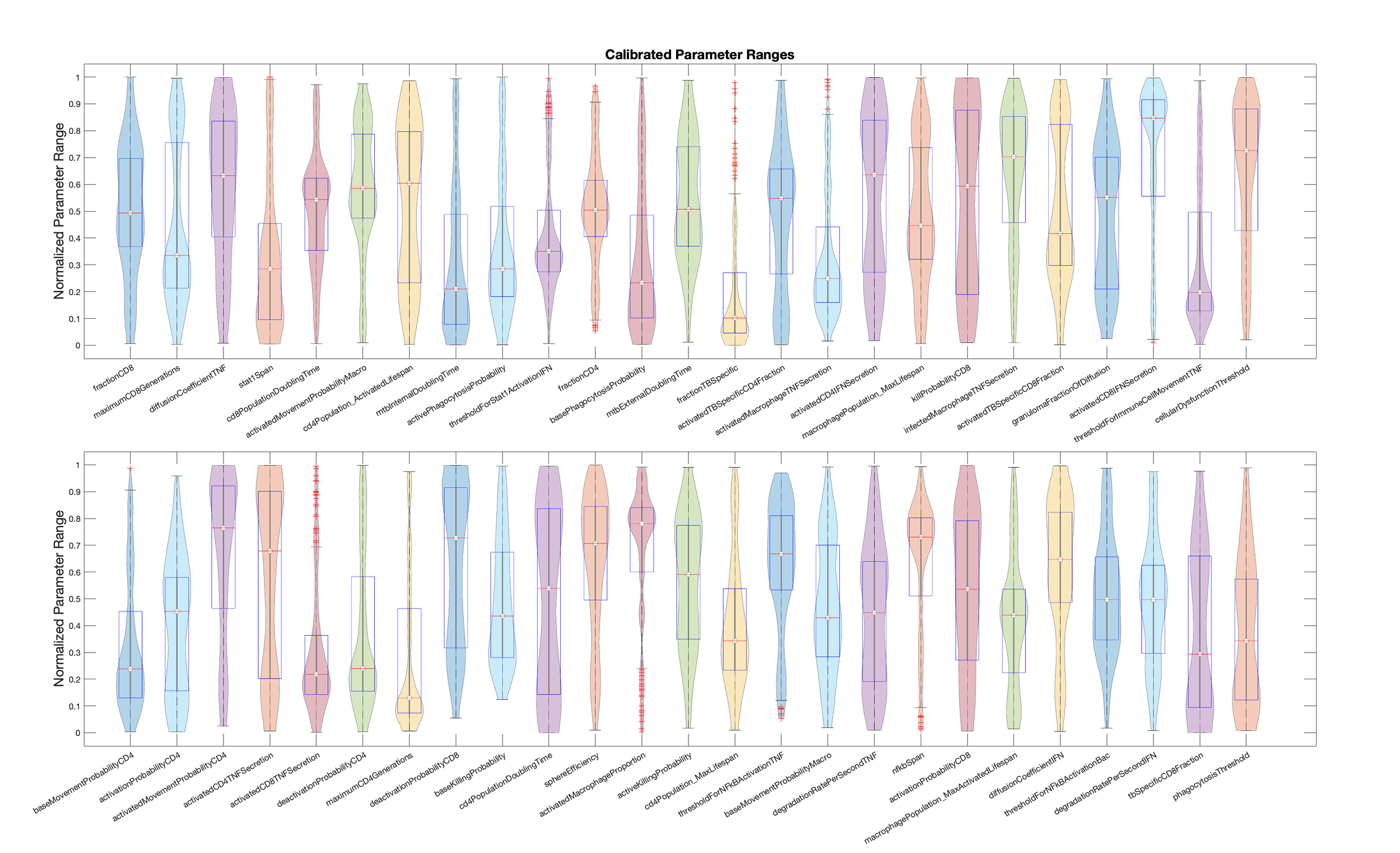

Supplement: S1 Fig — The ranges of the parameters have been normalized from 0 to 1 with the bounds representing the minimum and maximum of the ranges listed in Table 1. (TIF) [file pone.0299107.s001.tif]

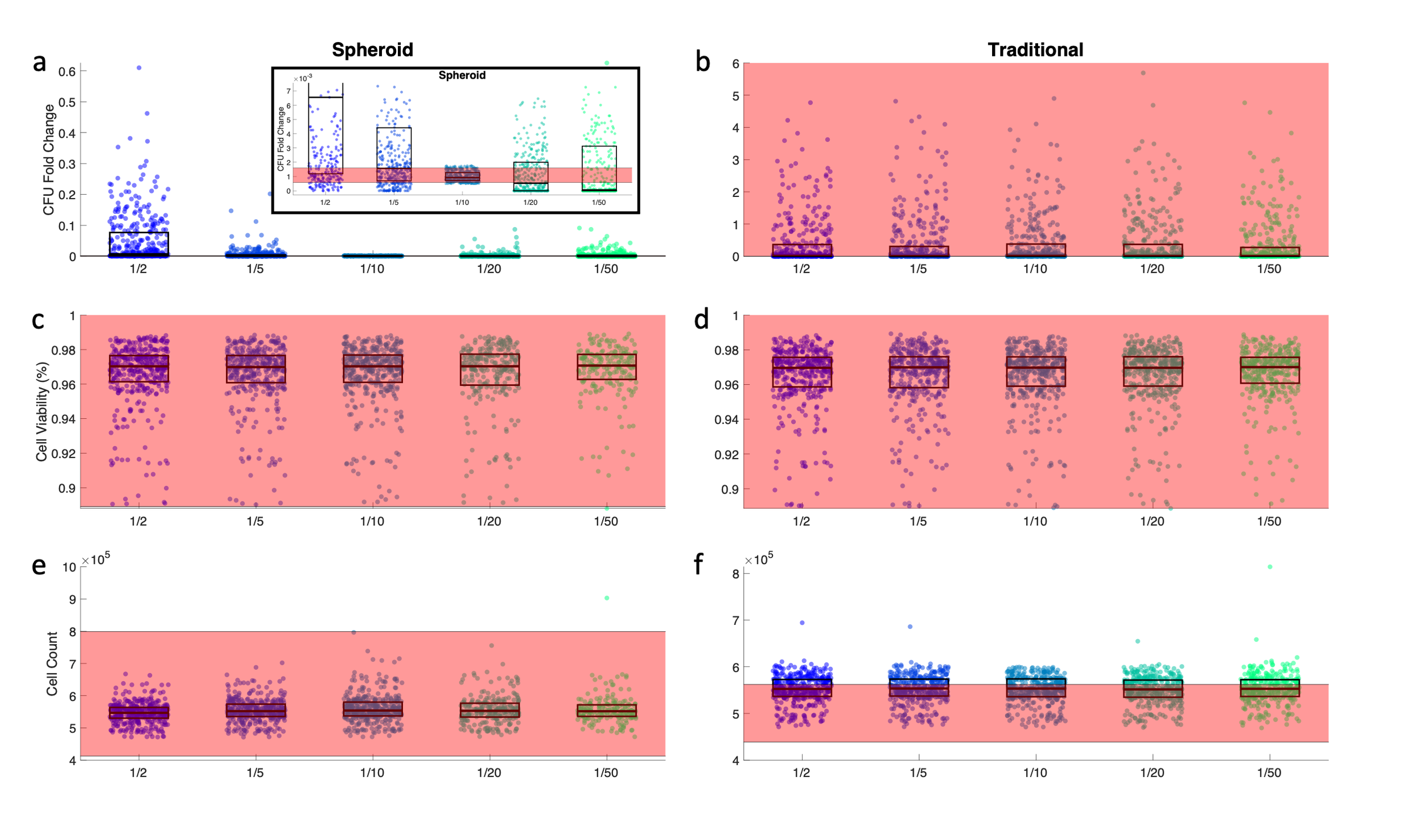

Supplement: S2 Fig — Spheroids that have been scaled to 1/2, 1/5, 1/10 (original), 1/20, and 1/50 size of the experimental culture were simulated using the calibrated parameters. Outputs for the scaled spheroid and traditional simulations were compared with the 6 outputs used for calibration: spheroid CFU change with zoomed y-axis (a), traditional CFU fold change (b), spheroid cell viability (c), traditional cell viability (d), spheroid cell count multiplied by one over scaling factor (e), and traditional cell count multiplied by one over scaling factor (f). Red regions represent the experimental ranges. As expected, the outcomes of the traditional simulation are similar regardless of how much it was downscaled. The spheroid simulation showed similar outcomes for normalized cell count and percent viability, but CFU fold change varied. Smaller simulated spheroid had lower CFU fold changes suggesting they are better able to control bacteria. These dots represent 398 runs, but one run is missing from the 1/2 spheroid simulation population and three runs are missing from the 1/2 traditional simulation population due to these runs exceeding wall time limits with maximum memory and time allocated. (TIF) [file pone.0299107.s002.tif]

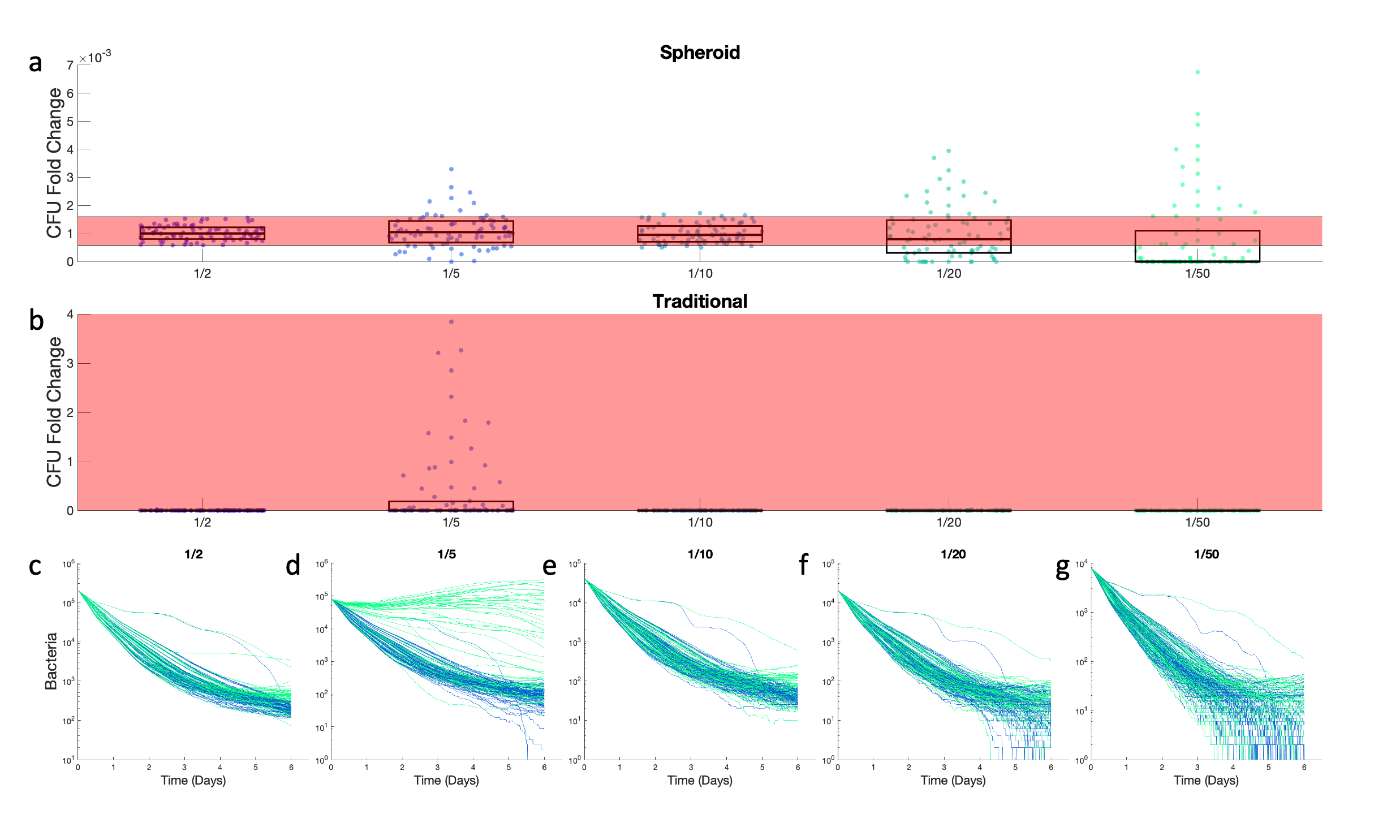

Supplement: S3 Fig — Subset of simulations that fall within spheroid and traditional CFU fold changes for both 1/2 and 1/10 scaled runs. CFU fold changes for spheroid simulations (a) and traditional simulations (b). Red regions represent experimental ranges. Time courses of bacteria count for traditional (lighter green) and spheroid (darker blue) show similar dynamics across scales (c-g). (TIF) [file pone.0299107.s003.tif]

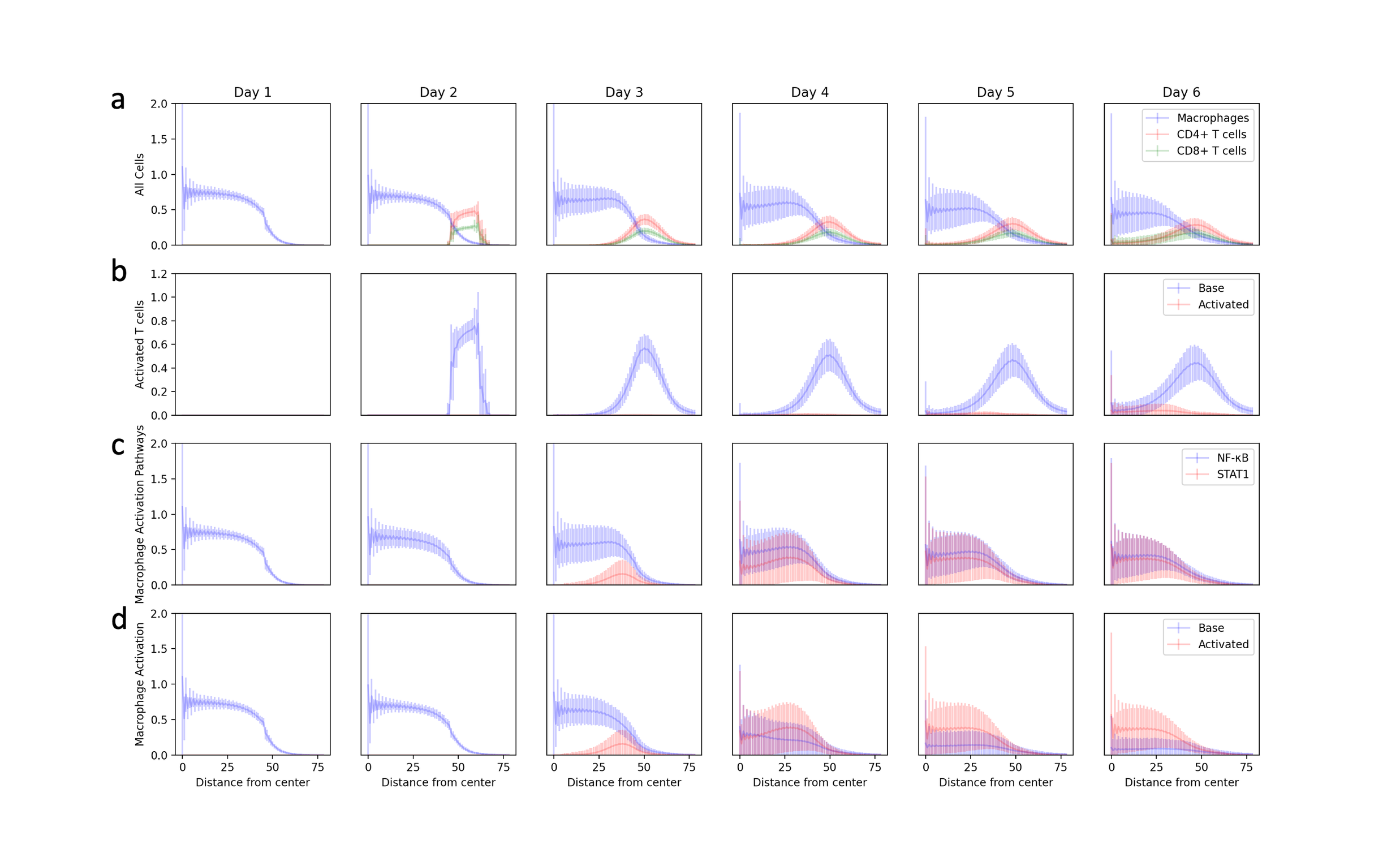

Supplement: S4 Fig — The radial distribution of a) macrophages, CD4+ T cells, and CD8+ T cells; b) base and activated T cells; c) NF-κB and STAT1 activated macrophages; d) base and activated macrophages. The y-axes represent the radial density of cells, which is calculated by number of cells at a given distance from the center of a spheroid divided by the volume of the spherical shell. All runs have been averaged with error bars representing standard deviation. (TIF) [file pone.0299107.s004.tif]
