# Supplementary material for: In silico agent-based modeling approach to characterize multiple in vitro tuberculosis infection models
Source: PLoS One. 2024 Mar 22;19(3):e0299107. doi: 10.1371/journal.pone.0299107 (PMC10959380; doi:10.1371/journal.pone.0299107)
Supplement: S5 Fig — After initialization, the simulation consists of a loop of agent secretion, diffusion, agent behaviors, and an agent watcher. Overview of actions is shown, and more detail can be found in the Repast model code at https://github.itap.purdue.edu/ElsjePienaarGroup/TB-in-vitro release v1.0.1. (PDF) [file pone.0299107.s005.pdf]

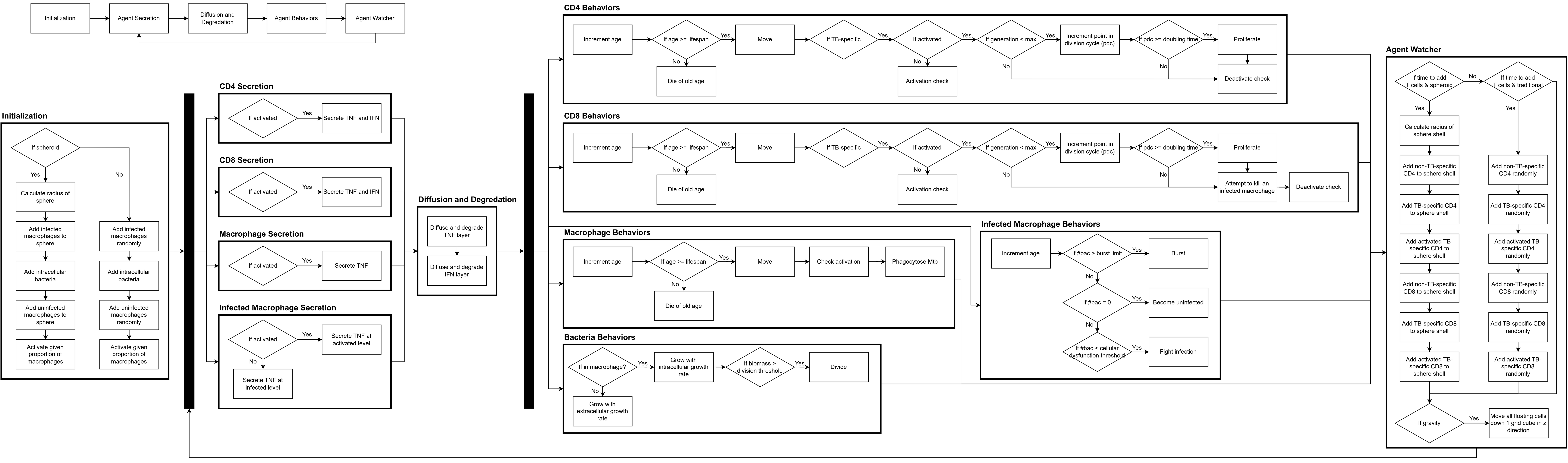

Initialization
Agent Secretion
Diffusion and Degredation
Agent Behaviors
Agent Watcher
Initialization
If spheroid
Yes
No
Calculate radius of sphere
Add infected macrophages to sphere
Add infected macrophages randomly
Add intracellular bacteria
Add intracellular bacteria
Add uninfected macrophages to sphere
Add uninfected macrophages randomly
Activate given proportion of macrophages
Activate given proportion of macrophages
CD4 Secretion
If activated
Yes
Secrete TNF and IFN
CD8 Secretion
If activated
Yes
Secrete TNF and IFN
Macrophage Secretion
If activated
Yes
Secrete TNF
Infected Macrophage Secretion
If activated
Yes
Secrete TNF at activated level
No
Secrete TNF at infected level
Diffusion and Degredation
Diffuse and degrade TNF layer
Diffuse and degrade IFN layer
CD4 Behaviors
Increment age
If age >= lifespan
Yes
No
Die of old age
Move
If TB-specific
Yes
If activated
Yes
No
Activation check
If generation < max
Yes
No
Increment point in division cycle (pdc)
If pdc >= doubling time
Yes
No
Proliferate
Deactivate check
CD8 Behaviors
Increment age
If age >= lifespan
Yes
No
Die of old age
Move
If TB-specific
Yes
If activated
Yes
No
Activation check
If generation < max
Yes
No
Increment point in division cycle (pdc)
If pdc >= doubling time
Yes
No
Proliferate
Attempt to kill an infected macrophage
Deactivate check
Macrophage Behaviors
Increment age
If age >= lifespan
Yes
No
Die of old age
Move
Check activation
Phagocytose Mtb
Infected Macrophage Behaviors
Increment age
If #bac > burst limit
Yes
Burst
No
If #bac = 0
Yes
Become uninfected
No
If #bac < cellular dysfunction threshold
Yes
Fight infection
Bacteria Behaviors
If in macrophage?
Yes
Grow with intracellular growth rate
If biomass > division threshold
Yes
Divide
No
Grow with extracellular growth rate
Agent Watcher
If time to add T cells & spheroid
No
If time to add T cells & traditional
Yes
Yes
Calculate radius of sphere shell
Add non-TB-specific CD4 to sphere shell
Add non-TB-specific CD4 randomly
Add TB-specific CD4 to sphere shell
Add TB-specific CD4 randomly
Add activated TB-specific CD4 to sphere shell
Add activated TB-specific CD4 randomly
Add non-TB-specific CD8 to sphere shell
Add non-TB-specific CD8 randomly
Add TB-specific CD8 to sphere shell
Add TB-specific CD8 randomly
Add activated TB-specific CD8 to sphere shell
Add activated TB-specific CD8 randomly
If gravity
Yes
Move all floating cells down 1 grid cube in z direction
