# Supplementary material for: In silico agent-based modeling approach to characterize multiple in vitro tuberculosis infection models
Source: PLoS One. 2024 Mar 22;19(3):e0299107. doi: 10.1371/journal.pone.0299107 (PMC10959380; doi:10.1371/journal.pone.0299107)
Supplement: S1 File — (DOCX) [file pone.0299107.s006.docx]

**Supplementary Information**

**S1 Table. Constant parameters.** Parameters that were held constant during sampling, their values, and units.

| **Parameter** | **Constant value** | **Units** |
| --- | --- | --- |
| **Traditional v Spheroid** | | |
| case number | Spheroid: 9  Traditional: 15 | - |
| is spheroid | Spheroid: 1  Traditional: 0 | - |
| grid dim X Y | Spheroid: 80  Traditional: 216 | Grid squares |
| grid dim Z | Spheroid: 80  Traditional: 11 | Grid squares |
| **Simulation defined** | | |
| is batch run | 1 | - |
| cells needed to be added to gran | 8 | Neighboring immune Cells |
| is plain colors | 6 | - |
| gran qualification immune cell count | 27 | Immune cells |
| random seed | Random | - |
| diffusion time step multiplier | 4 | - |
| time step | 6 | Minutes/step |
| agent limit | 60000000 | Agents |
| cells needed to remain in gran | 8 | Neighboring immune cells |
| division biomass threshold | 2 | - |
| **Experimentally defined** | | |
| days to run | 6 | Days |
| fraction CD3 | 1 | CD3+ cells/lymphocytes |
| initial PBMCs | 100000 | Cells |
| fraction monocyte | 0.4 | Monocyte/PBMC |
| fraction lymphocyte | 0.6 | Lympocyte/PBMC |
| time to add T cells | 48 | Hours |
| **Variance** | | |
| CD4 doubling time variance  CD8 doubling time variance | 0.25 |  |
| NF-κB variance | 0.1 |  |
| *Mtb* growth rate variance | 0.1 |  |
| STAT1 variance | 0.1 |  |
| macrophage lifespan variance | 0.1 |  |
| CD4 lifespan variance  CD8 lifespan variance | 0.1 |  |
| division biomass variance | 0.2 |  |
| new *Mtb* placement range | 0.2 |  |
| min burst limit | 20 | Internal bacteria (41) |
| max burst limit | 40 | Internal bacteria (41) |


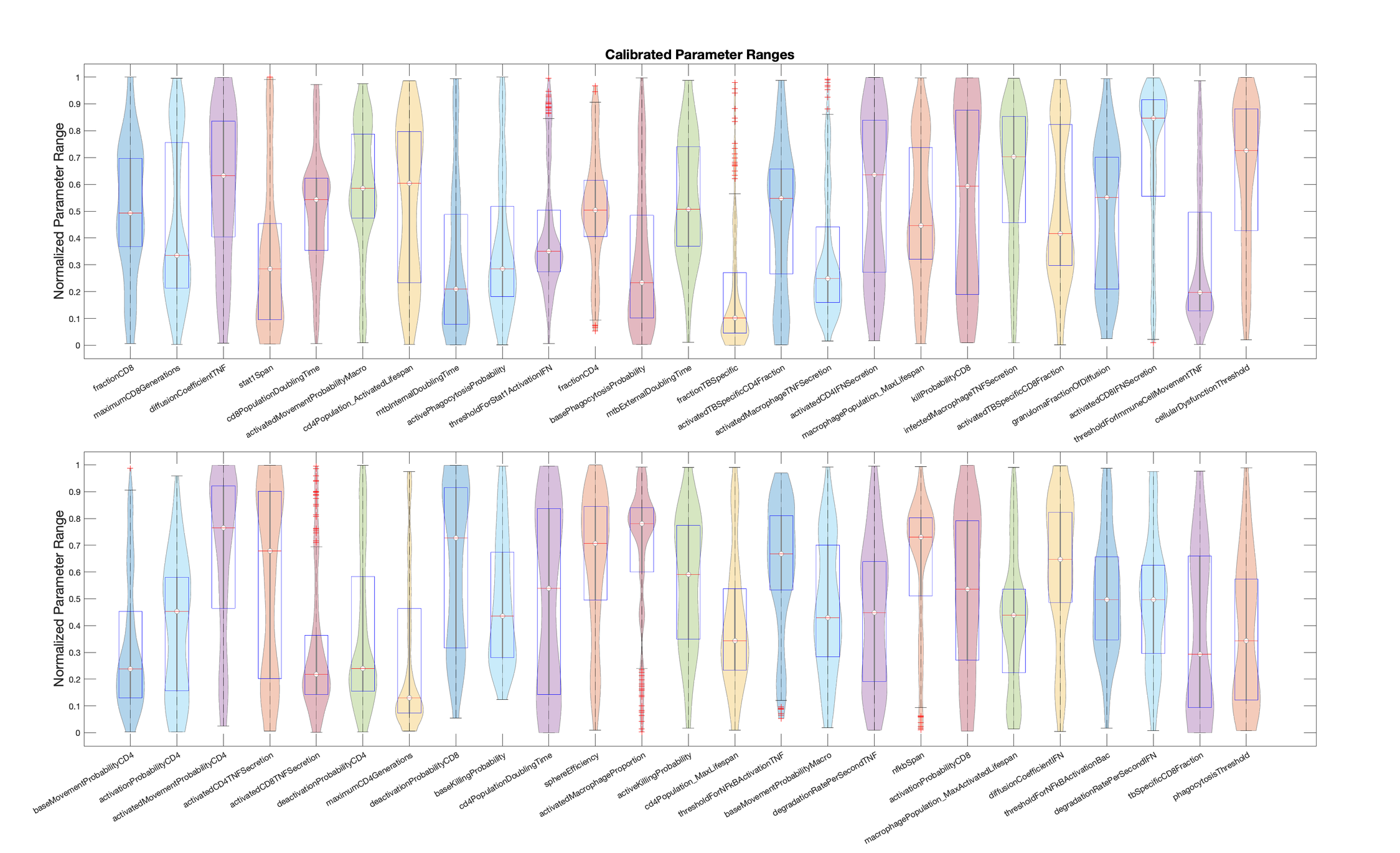


**S1 Fig. Distribution of parameters in calibrated runs.** The ranges of the parameters have been normalized from 0 to 1 with the bounds representing the minimum and maximum of the ranges listed in *Table 1*.

**S2 Fig.** **Scaled-up simulations.** Spheroids that have been scaled to 1/2, 1/5, 1/10 (original), 1/20, and 1/50 size of the experimental culture were simulated using the calibrated parameters. Outputs for the scaled spheroid and traditional simulations were compared with the 6 outputs used for calibration: spheroid CFU change with zoomed y-axis (a), traditional CFU fold change (b), spheroid cell viability (c), traditional cell viability (d), spheroid cell count multiplied by one over scaling factor (e), and traditional cell count multiplied by one over scaling factor (f). Red regions represent the experimental ranges. As expected, the outcomes of the traditional simulation are similar regardless of how much it was downscaled. The spheroid simulation showed similar outcomes for normalized cell count and percent viability, but CFU fold change varied. Smaller simulated spheroid had lower CFU fold changes suggesting they are better able to control bacteria. These dots represent 398 runs, but one run is missing from the 1/2 spheroid simulation population and three runs are missing from the 1/2 traditional simulation population due to these runs exceeding wall time limits with maximum memory and time allocated.

**S3 Fig. Scaled-up simulations that maintain CFU fold changes.** Subset of simulations that fall within spheroid and traditional CFU fold changes for both 1/2 and 1/10 scaled runs. CFU fold changes for spheroid simulations (a) and traditional simulations (b). Red regions represent experimental ranges. Time courses of bacteria count for traditional (lighter green) and spheroid (darker blue) show similar dynamics across scales (c-g).

**S4 Fig. Average radial distribution.** The radial distribution of a) macrophages, CD4+ T cells, and CD8+ T cells; b) base and activated T cells; c) NF-κB and STAT1 activated macrophages; d) base and activated macrophages. The y-axes represent the radial density of cells, which is calculated by number of cells at a given distance from the center of a spheroid divided by the volume of the spherical shell. All runs have been averaged with error bars representing standard deviation.

**S5 Fig. Flowchart of the simulation.** After initialization, the simulation consists of a loop of agent secretion, diffusion, agent behaviors, and an agent watcher. Overview of actions is shown, and more detail can be found in the Repast model code at https://github.itap.purdue.edu/ElsjePienaarGroup/TB-in-vitro release v1.0.1.
