# Supplementary material for: In silico agent-based modeling approach to characterize multiple in vitro tuberculosis infection models
Source: PLoS One. 2024 Mar 22;19(3):e0299107. doi: 10.1371/journal.pone.0299107 (PMC10959380; doi:10.1371/journal.pone.0299107)
Supplement: Attachment — Submitted filename: 2023-07-23PLOS_Response.pdf [file pone.0299107.s007.pdf]

We thank the reviewers and the editor for their thoughtful and thorough feedback. Below, we respond to each reviewer's comments, and we provide a highlighted copy of the edited manuscript.

Editors Comments:

Please bear in mind that the objective of modeling studies such as yours is not only to generate simulations that approximate observed behavior; it is to make testable predictions. While I commend your attempts to explore parameter sensitivity, with such a large model one inevitably runs a risk of over-specification. Quantitative out-of-sample predictions are essential for testing your model's utility.

Thank you for this constructive high-level feedback. We now include explicit discussions of our out-of-sample predictions based on calibrated model results to each of the results sections (Lines 544-551; 557-558; 603-609; 673-677; 755-764; 804-807).

The manuscript contains many instances of descriptions of your model as a tool. Please maintain focus on the specific advances that your study made.
In addition to the changes made in response to the previous comment, we have edited the manuscript as a whole to shift the focus from model use and potential to the biological insights that we gained from our simulations. Specifically, we removed references to potential tool applications in the abstract, introduction, and results sections; removed uses of 'can be used' and instead emphasize the work done here and the predictions we make.

"You could also remove the more general statements regarding the value of modeling, which are unnecessary for Plos Computational Biology. Doing so will also make it more concise."
We removed the paragraph about benefits of computational modeling in the introduction.

It would be useful to give a more detailed overview of what validated insights large-scale models of TB have given us, and how your model fits into that body of work.
We now include referenced reviews of contributions of large-scale models of TB to understanding of disease in introduction and highlighted two validated examples. We also emphasized that our model represents *in vitro* systems thereby complementing findings from *in vivo* systems as previous models.

"One other minor point is that zebrafish are mentioned as a model organism later in the paper but are not mentioned in the list in the introduction."
Zebrafish are now added to the list in the introduction. Benefits and drawbacks of this model organism were also included.

Reviewer #1 Comments:

In this paper, the authors introduce a novel framework to simulate various in vitro tuberculosis infection models (spheroids and traditional cell cultures) computationally using an agent-based model. The paper is very well-written and provides a powerful tool to better understand and analyze in vitro experiments mechanistically. This paper connects in vitro work on TB with and ABM to make predictions about outcomes in different settings. The goal of designing an *in vitro* spheroid culture model is well motivated to contextualize properties of traditional culture against new spheroid culture techniques to investigate the disease of TB, as well as allow virtual studies not possible with *in vitro* spheroid cultures. There are also two exciting results that stood out: Figure 3 provides radial compositions of cell composition and interactions from the center of the granuloma to the lymphocytic cuff that are of increasing immunological interest with *in vivo* models but such experimental models are statistically limited by analysis of the center slice of the granuloma. Additionally, the observation of slower doubling time of internalized bacteria early in infection leading to more total bacteria output is fascinating: it supports the literature of stressed bacteria arresting their replication and metabolism for persistence.

Major comments:

1) Maybe a little more background on in vitro granulomas, traditional in vitro and spheroids and exactly what they are and the 2-D versus 3D stuff is a little vague to me. Also how are they useful beyond animal models like mouse and NHP? When are they used?

The relevance of in vitro vs in vivo granulomas, is the ability to use this model as a tool to investigate the functional implications of targeting specific genes/molecules/pathways, etc. In general, we consider this a tool for mechanistic investigations that might not be feasible in the in vivo context. We now include a short description of the in vitro models and their use relative to in vivo models (lines 101-107).

2) This paper focuses on cellular granulomas. Although mentioned briefly in Introduction (L 88), the authors haven't elaborated on necrotic granulomas in the paper. Caseous/Necrotic granulomas are very relevant to NHP and human granulomas, and they are the main driver of active disease. My understanding is that spheroids and cell cultures do not have necrotic tissue, thus the model does not simulate it. However, I believe it would be worth mentioning and discussing as a limitation.

This is an important point. We have now added discussion of necrotic granulomas into limitations (lines 862-872). We also emphasized findings from the *in vitro* paper that show beginning of necrosis. Both necrotic and nonnecrotic alveolar macrophages were seen in the center of the spheroid culture, as shown with the cytoplasmic localization of HMGB1. Experimentally induced hypoxia was necessary to produce the same results in the traditional culture, but not needed to induce these conditions in the spheroid.

3) The abstract mentions that in vitro models are useful for drug screening (L 28-29). However, the authors did not talk about the use of these in vitro and in silico models on drug studies anywhere else in the paper. I think it would be useful to mention how this

model would contribute to TB drug studies. The main challenge with drug studies is penetrating into the necrotic core of granulomas. Since this model does not include necrotic core, this may be a limitation for drug studies too. However, I believe that this model may give insight into treating cellular granulomas.

We now include a description of how drug screening could be interpreted for these in vitro models (lines 758-767). We also added that drug screening is limited to cellular and early necrotic granulomas (lines 862-872).

4) L351: What corrections are applied when simulating a spheroid 1/10th the size? Distance, parameter corrections, etc? Is the *sphereEfficiency* term mentioned on L357 involved in the correction?

One tenth size was selected due to computational limitations, and the fit parameters were assumed to be effective parameters. We have however run representative simulations up to a ½ scale spheroids. We simulated 1/2, 1/5, 1/10 (original), 1/20, and 1/50 sized spheroid using the calibrated parameters and compared the 6 outputs that we used for calibration. As expected, the outcomes of the traditional simulation are similar regardless of how much it was downscaled. The spheroid simulation showed similar outcomes for normalized cell count and percent viability, but CFU fold change varied. Smaller simulated spheroids had lower CFU fold changes on average suggesting they are better able to control bacteria for the same parameters. This could be a good starting point if recalibration at a higher scale was performed. This analysis was added to the supplement (Figure S2) along with an acknowledgment to the OSG resources used to perform this analysis.

5) In the model construction, it is explicitly mentioned in a few cases (e.g. *populationLifespan* combined with *lifeSpanVariance*) that there are parameters that are varied between cells of the same type within a population. Does this suggest that other parameters are identical between all cells in the population? If this is the case, perhaps the authors may briefly discuss what implications they would expect to find from representing within-population cell heterogeneity in a more detailed manner, and what importance these model construction choices may hold.

We now added parameter sampling details to section 2.2.4 to clarify which parameters have variance and how it was included. We also added discussion on the implication of parameter variance to limitation section (lines 925-936).

MINOR comments:

"How the calibration sweeps are performed need a little more clarity in methods."

We now include a diagram of simplified calibration iterations, which is included as Figure 3.

"I think a methods figure cartoon with 3 plates explaining the in silico the in vitro and the spheroid schematically would help. It could also be a layer above f,g,h in Figure 2."

We now added figure 1 to explain the four conditions (spheroid experimental, traditional experimental, spheroid simulation, and traditional simulation). These labels are mirrored in an edit on Figure 3.

“Rationale for why certain cells and cytokines were included and not included is needed.”
We added justification for selection of TNF and IFN to section 2.2.1. And added justification for cell type selections to section 2.2.2.

“In methods..is an “internal bacterium” an intracellular bacterium? I would use that language.”
All reference to internal bacteria were changed to intracellular.

“should define Moore nbhd.”
Added a definition of Moore neighborhood to the first occurrence.

“This sentence (line 430) “output spaces that are not accessible using the paired simulations” Needs clarification. It is jargony and unclear what it means.”
We changed this language (lines 774-779) to directly say that we are looking at traditional runs with high cfu, something that was unachievable using paired runs.

“hpi is never defined.”
Replaced hpi with hours post-infection.

“In some of the figures it is confusing whether the data presented are simulated or experimental. That needs clarifying throughout.”
We apologize for this confusion. We now added Figure 1 to assist in clarifying experimental and simulated groups. Added labels to 3, to mirror setup in Figure 1 and made language consistent throughout to clarify simulations vs experiments.

“FIGURE 6 is just toooo small!! I blew it up 200% and then it was blurry.”
We now split panels a and b onto separate lines to allow panels to be larger.

“Could shorten intro paragraph on why computational models are great (pp 5-6), given the journal”
We have removed the paragraph about benefits of computational modeling in the introduction.

Recommendation wrt style - end the intro with something more motivating than “finally, we use”.
We hanged the final sentence to include motivation of looking at differences between traditional and spheroid models.

“The decision to use TNF and IFN (or “like”) is not well motivated, should mention in intro or else in section 2.2.1. Reader doesn’t know if there are many many cytokines, or really just these two, or why these two in particular are important to capture (e.g. their pro- and anti-features)”

We added justification for selection of TNF and IFN to section 2.2.1.

I thought the bottom of page 28 was interesting - two different ways to control the bacterial - but it was pretty hidden there. Similarly, the conclusions around line 655 were interesting but hidden. The paper might be more impactful if some higher level conclusions could be pulled out, e.g. around drug screening.
Thank you for highlighting these findings that were buried in other discussions. We now highlight these findings explicitly in each results section (lines 673-677; 755-764).

L 181: Is there a particular reason why experimental data are collected at day 6? Is it an enough time for spheroids to form? Granuloma formation is a lot slower in vivo so it would be good to clarify this.
We have added this clarification to lines 188-191. The day-6 timepoint was selected as the timepoint whereby the structure first demonstrated good stability and the time whereby we expect to detect measureble T cell immune responsiveness. The stability was determined by verifying that the macrophage plus T cell rim remains intact despite removal of the magnet. In vivo, the lengthy time required for granuloma formation is in part due to the delay in T cell priming in the lymph node. This process is circumvented in the granuloma model through direct addition of T cells to the structure. This is beneficial because it allows us to study disease mechanisms within a shorter period of time, shortening the time to which potentially important outputs are identified. Longer incubation timepoints are being investigated, keeping host cell viability in consideration.

"Fig1b: Are resting macs and resting CD4+ T cells the only cell types that move? Are activated macs, activated CD4+ and CD8+ T cells the only cell types that die due to aging? It may be worth clarifying these."
We have now added other cells to Figure 1b to be more complete.

"Fig1d: This panel looks confusing, activation seems to be a part of panel c."
We adjusted panels to correspond to cell types and incorporated panel d into panel c.

"L348-349: It is not clear to me how the effect of levitation beads are included in the computational model."
We clarified that the effect of the levitation beads in included by changing the movement rules (lines 398-399).

"L 358: What is sphereEfficiency? I didn't see it defined anywhere."
We now defined sphereEfficiency (lines 383-386).

"L 369: A verb is missing in this sentence."
We added a verb to this sentence.

"L 391: What is hpi?"
We replaced hpi with hours post-infection.

“L 397-413: It would be better to describe this section with a diagram or figure that may make it easier to understand how the iterations work.”
We now include a diagram of simplified calibration iterations, which is included as Figure 3.

“Table 1: What is ‘per tick’?”
All references to tick were replaced with time step, which is equivalent to 6 minutes.

“L 435: Typing the cost function as an equation would be clearer.”
We now include an equation for the cost function.

“Fig2a-c: How many simulations/experimental data are there in the box plots? It would be better to show the dots for the experimental results as well.”
Made the dots for the experimental system larger so they don’t get covered by the box plot. Added n values to the caption.

“Fig4c: Legend is missing. How the runs are sorted and what the dots represent are not clear to me. Are they the mean or standard deviation?”
The runs are sorted by the mean minus one standard deviation (bottom of blue bar). We fixed the legend to include blue and red boxes and lines.

“L 782: GranSim runs start with one intracellular Mtb, not extracellular.”
This reference to initial extracellular bacteria was removed.

“L98: Maybe introduce what “tractable” means in this context of the experimental system? It seems that we’re still discussing experimental models here and using this term is not unfamiliar because it does not appear to relate to computational complexity. Certainly, the word is mentioned several times in the citation 12 of Elkington et al. (2019) J Infect Dis., but tractable is also not clearly defined there either. Perhaps a quick explanation of the word in the introduction may help?”
We added a sentence redefining tractable to introduction.

“L108: Not clear what “platforms” means here. This is the first use of the word. Is it referring to “[experimental] systems”? The next sentence uses the word “systems”, so maybe keep using the word for easier understanding despite the repetition.”
We changed platforms to experimental systems for consistency.

“L115: It may help to better separate the usage of “models” and [experimental] “systems” throughout the manuscript. It’s unclear what each is referring to.”
We changed system to in vitro model in the introduction.

L177: There seems to be a typo with “n3d”. On the Greiner website, they have a whitepaper for “m3d” (magnetic 3d culturing), not n3d: https://shop.gbo.com/en/usa/products/bioscience/3d-cell-culture/magnetic-3d-cell-culturing/657841.html

There has been some naming changes due to Greiner acquiring Nano3D (n3D) Biosciences. We have removed this reference to n3D.

"L216: The legend description of figure 1d is a little confusing: saying that macrophage activation is a "two-step" implies a sequential process, whereas figure 1d seems to indicate 2 alternate paths to the same outcome as a non-sequential "either-or" process. Both are required according to L267, therefore consider adding a note to figure 1d near the activated macrophage indicating that both activations are required."
We added note to Figure 1 macrophage panel and changed the caption to clarify that the process is non-sequential.

"L354: Seems disconnected from the preceding sentence that provides rationale for the formula. Consider joining the 2 sentences: "...phagocytosed bacteria(45), giving us the fraction of macrophages that have phagocytosed *n* bacteria as $\frac{MOI^{n}e^{-MOI}}{n!}$.""
These sentences were combined as suggested.

"L374: Does keeping the ratio constant between the experimental system and the simulation involve using a forcing function of experimental ratios being applied to simulation ratios at each time point? If so, it may be useful to know how often that ratio is sampled. Or is the ratio a single constant value from the experimental system applied to the simulation for all timepoints? Either way, it may help to clarify in the manuscript."
We clarified that this ratio is only used to initialize the system.

"L397: It's a little confusing to read the number of parameters varied in the model in the calibration section, because the number of parameters varied for a simulation can be different from the number of parameters varied during calibration. Please confirm whether the 50 parameters were varied only for calibration as mentioned in Table 1, or perhaps move lines L397-L399 into a model and sampling section before the calibration section with any additional detail."
We now added detail about parameter sampling between cells and clarified that the calibrated parameter set has all 50 parameters varied.

"L414: Please clarify if all the parameters in Table 1 assume a uniform distribution or if any parameters assume any other type of distribution. Please also indicate if any parameters are discrete (such as *maximumCD{4,8}Generations*)."
We clarified that all parameters are sampled in uniform distributions and added that all parameters are continuous. Note, that 4 parameters behave discretely as they are only used in comparison to integer values.

"L417: There are 50 rows of parameters, but 54 parameters when including the last 4 rows in the $CD4^+$ T cells. Why are they called 50 parameters when there are 54? Are those values of the $CD4^+$ and $CD8^+$ cells made identical? If so, please explain this in the Table 1 caption."
We clarified that these four parameters are shared between CD4+ and CD8+ T cell populations.

“L442: Figure 2b either the y-axis label should include % for units of cell viability, or the y-axis should be in the range of 0.8-1.0.”
We added (%) to y-axis label.

“L486: I wonder if “bulk” could more precisely be called “aggregate” or “count” data? This is the first use, and is later used on lines L756 and L759. The next line uses the word “cell counts”.”
References to “bulk” data were changed to aggregate data.

“L494: Can confidence bands for the cells be provided across simulations in Figure 2b-e? Maybe as a supplementary figure, if the plots would be too busy to overlap multiple cell types in the main manuscript. For example, see the radial plot in Figure 5d of 10.1111/imr.12671 with the large amount of variation.”
We added this suggested figure to the supplement.

“L497: Consider briefly recapitulating the statistical normalization of the y-axes in the figure caption that is detailed in L509-L514.”
We now added a description of y-axes measure/radial density to the caption for Figure 3.

“L527: Is it possible to confirm and quantify from the simulations that indeed TNF-α is driving the NF-κB activation? Not asking for a figure; simply to better support the sentence in the text, and it would be a useful finding.”
In our simulations, the NF-κB activation is solely due to TNF for the first two days. We added this detail to the text.

“L531: It’s hard to get a sense of the infiltration from the point graph without knowing the overall size of the granuloma. Maybe a dotted line indicating the granuloma size for the simulations would be helpful? Or normalize to the granuloma size and plot the percentage infiltration on the y-axis, especially because T cell infiltration is described in section 3.3. To preserve the absolute information in figure 4c, the corresponding percentage infiltration y-axis could be an additional figure 4d which may or may not require a different x-axis sorting method.”
The point graph was supposed to include bars for the standard deviation, but this was lost in formatting during submission. We now added panel d that normalizes to the initial size of the macrophage spheroid with a line for initial size of full spheroid though.

“L531: There appears to be a graphical plotting but in the legend of Figure 4c; the blue and red colored squares for the T cells and Macrophages do not appear in the legend. Presumably red represents macrophages and blue represents T cells.”
We fixed the legend to include blue and red boxes and lines.

“L531: In this Figure 4 and the preceding figures, there are 2 numbers in white inscribed in the simulation 3D plots “1440.0” and “177” / “235”. Such numbers are also present in Figures 2 and 3. From figure 3, the top-right number seems to be the time step. The bottom-left number is

unclear and seems to vary from 27–235. Maybe this set of numbers could be described in one of the figure captions somewhere — maybe figure 2 — to satisfy the curious reader?"
We added descriptions of these numbers to the Figure 2 caption.

"L573: Maybe the topic position of the first sentence of the paragraph could introduce this paragraph as relating to granuloma structure / spatial layout of cells?"
We added a topic sentence to this paragraph that relates spatial layout of cells to activation status.

"L585: Consider braces or vertical bars on the right of the Figure 5 y-axis labels of parameter groups to explicitly delimit the parameter names. Alternatively one could use horizontal lines for each group that crosses through the plot heatmaps. *randomSeed* should be separated from diffusion."
We corrected an error that removed gray bars running across parameters, ensured randomSeed was not in diffusion category

"L600: The literature documents how arresting replication and metabolism is a stress-adaptive function of *Mtb*, and observing this additional connection lower replication increasing total CFU appears to support this broad strategy of no/slow-replication persistence. However, from a mathematical perspective, this is a curious and not an intuitive observation. The lower doubling time leading to more generations and to more bacteria may be better supported here by restating or elaborating the driving parameters of internalized bacterial killing before day 2. L284 states that an internal bacterium is selected randomly and killed; my naïve interpretation is this is a somewhat uniform rate of killing independent of the bacterial doubling time. There isn't any equation in the manuscript or supplement that helps alleviate this confusion. It's also perfectly fine to address this in future work, but either way some clarification would help."
Clarified that smaller doubling time is equivalent to faster growth rates. Added the simplification of bacterial phenotype to discussion.

"L609: Consider restating the other output introduced 2 paragraphs ago: "... is the only other output, besides total bacterial count, showing significant correlations …""
We restated the other output as suggested.

"L654: Maybe reference the macrophage lifespans and activation in Figure 5 and/or use their parameter names?"
We added parameter names for macrophage lifespan parameters to the text.

"L707: Please join with the previous sentence; initially upon reading the sentence, "This model" made me think of the author's model, but on rereading it seems to instead refer to the "network model of tumor cell infection …""
These sentences were combined as suggested.

"In Figure 2-D: there seems to be a sharp dropoff around day 3.5ish. Is there anything interesting happening there?"

This is an excellent observation, and it will be addressed in a follow up manuscript currently in preparation. The dynamics leading to the drop-off are complex and would have made this manuscript too long.

“When your cells divide, what happens if they don’t have room? Do they push other cells away from themselves, or is there some mechanical or nutritional inhibition that prevents them from growing if the grid is saturated?”
We added detail about failed generations when grid is saturated, along with percent of attempted divisions that fail.

Line 57 - How might “users” tell which model to choose over the other from your model output? Is this just from biological consideration, qualitative result, etc.?
We added more example considerations for use cases to section 3.4.

Line 110 - Could you say a few words about why it would be reasonable to synthesize “disparate system” results? Is the biology of the system similar enough to rule out interactions from simplified and disparate *in vitro* systems? The authors should expand a bit or include some citations.
We added a couple of sentences to the introduction in the *in silico* paragraph to expand on how information could be synthesized and how it would be informative even if it didn’t work.

“There’s a little bit of inconsistency in the camelCase used for variables. Some are written as lowerUpper and some as UpperUpper. Moreover, in the SI, there is a part of the table with “unused parameters”. While I can imagine why these are here from a modeler’s perspective, they kind of get in the way of understanding the exposition even if it’s likely helpful as an internal document. I’d recommend making the capitalization consistent and remove references to variables that aren’t used, mentioned, or are for other models (e.g. “timeToAddInfectedCells = -1” and “virusDiffusionCoefficient = 0”).”
We removed unused parameters from SI table. We also adjusted camelCase of parameters throughout document and figures to be consistently lowerUpper.

Reviewer #2 Comments:

The remainder of this manuscript takes it as demonstrated that the in-silico system recreates the in-vitro one, and discusses details that are not experimentally measurable.
We now include explicit examples of how our predictions could be tested with further experiments (Lines 607-609; 637-639).

There are many sentences about what a model "can" do, not helpful to the readers of PLoS Computational Biology who will already be convinced.
We have removed most references to what the model ‘can’ do, and instead focuse on what we have done. As mentioned in our response to the editors comment we have edited the

manuscript as a whole to shift the focus from model use and potential to the biological insights that we gained from our simulations. Specifically, we removed references to potential tool applications in the abstract, introduction, and results sections; removed uses of 'can be used' and instead emphasize the work done here and the predictions we make.

"More interesting to the readers would have been an investigation of the formation of the structure."
This structure is created by hand, so the initial formation is dictated by experimental setup. Immunological investigations into the chemokine gradients and mediators involved in the chemotaxis of T cells towards the infected macrophages, are planned as part of future work.

"What happens when there are fewer initial cells?"
When simulating smaller granulomas with the same parameters, we see they have lower CFU fold changes. This is now detailed in the supplement.

Is there a characteristic size of a granuloma that emerges from the diffusivity of molecules or is the spheroid essentially created by hand?
This structure is created by hand, so the initial formation is dictated by experimental setup. For each experimental well, the same proportion of both alveolar macrophages ($6x10^5$) and T cells ($4x10^5$) are added, with a total of $1x10^6$ cells per well. This resulted in structures which measured approximately 2.1mm in diameter.

How long does it take to form a spheroid and what is its ultimate fate (in silico predictions that could be tested in vitro)?
The spheroid cores form in approximately 24 hours. Indeed longitudinal assessments will allow for assessment of "complete destruction" of the structure by uncontrolled Mtb infection, versus cell death leading to optimal killing of the pathogen. Such testing is part of the planned future work.

Where cells are initially positioned, how they move in silico and in vitro (and typically how much over 6 days) is not clearly stated.
In silico the cells are initially positioned in a sphere in the center of the simulation space, and the movement occurs according to the rules described in the methods section.
In vitro, macrophages ($6x10^5$ total macrophages) are positioned at the core using the magnetic levitation method. Once the core had been stably formed (after about 48 hours), unlabeled T cells were added directly above the macrophage core. Because the T cells were unlabeled, the T cell cuff formed naturally at the outer edge of the macrophage core by a chemotactic gradient. The core measured ~1.9mm in diameter, with the T cell cuff ranging from 171μm – 310μm in diameter. T cell infiltration into the core was not readily observed, but the deepest infiltration of T cells within the core was measured at 97μm deep.

"Are two molecular species necessary? What are their diffusivities?"

We added justification for selection of TNF and IFN to section 2.2.1. Diffusivities are found in Table 1.

Fundamentally for the readers: What is the simplest model that would produce something that resembles a granuloma? How does this 3D model differ, in essence, from published 3D models of tumours?
This is an interesting question, but outside the scope of this work. The simplest model would be bacteria and macrophages, with the addition of T cells being necessary to represent a granuloma past 2-4 weeks. While the structure is important in granulomas and tumors, the cellular makeup of the respective systems is quite different. Additionally, *in vivo* the tumor would form from one cell growing from the inside out, while a granuloma is formed through the recruitment of immune cells from the outside in.

Other changes
We corrected units of decay to 10^-4 1/s.
We updated Figure numbers and references to reflect the addition of two figures.
