# Supplementary material for: In silico agent-based modeling approach to characterize multiple in vitro tuberculosis infection models
Source: PLoS One. 2024 Mar 22;19(3):e0299107. doi: 10.1371/journal.pone.0299107 (PMC10959380; doi:10.1371/journal.pone.0299107)
Supplement: Attachment — Submitted filename: PLoS_TB_ABM_MV.pdf [file pone.0299107.s008.pdf]

In this paper the authors simulate *in vitro* Tb infection models, spheroids and traditional cell culture. They developed an ABM to compare the simulations from these models and understand the mechanistic differences between the framework using: uncertainty analysis. They demonstrate and present a qualitative comparison of their simulation results from the spheroid and traditional cultures to that of the experimental results. The paper is well written, however there are some points and sub figures that need clarity. Below are suggestions that can help enhance clarity.

- Authors are recommended to add what a monolayer/traditional culture in vitro model is comprised of. How are these different than spheroids both in terms of structure, constituents, and complexity.

- What is the maximum age of the cell and how it differs from death rate used otherwise. Per 2.2.4, maximum generations are held constant between all cells, does that relate to the age of the cells and the rates used?

- L293: What are the set durations for the NF-kB and STAT1 activation and how are these determined?

- L: 306: Explain what *within an infected macrophage* mean? Do the authors mean from the infected macrophage's Moore neighborhood?

- What is the threshold of intracellular bacteria in monolayer versus spheroid structures, are these assumed to be the same i.e. 20-40 from a uniform distribution?

- For Fig 4, to perform a head on comparison to '4f', does 4g time step of 6min correspond to day 6? Please explain.

- Define what sphere efficiency is and the formula that defines it.

- What do 173 and 233 in Fig 6a represent? Are these the number of cells at Day 6?

- L 755-756: Why do T cell addition lead to diversion in influential parameters in traditional versus spheroidal? Can the authors suggest a hypothesis if possible?

- The legend for Fig 8b looks similar for spheroid and traditional, please fix or clarify.
