# Supplementary material for: In silico agent-based modeling approach to characterize multiple in vitro tuberculosis infection models
Source: PLoS One. 2024 Mar 22;19(3):e0299107. doi: 10.1371/journal.pone.0299107 (PMC10959380; doi:10.1371/journal.pone.0299107)
Supplement: Attachment — Submitted filename: 2024_01_30_ResponseToReviewer.docx [file pone.0299107.s010.docx]

 “Authors have provided a link to the GitHub repository however the ReadMe files are not updated with instructions on how to run the code, and recreate the figures. Authors are recommended to document the ReadMe file in the GitHub repository and include instructions to help re-create the figures.”

Thanks for this suggestion. The GitHub holds the code to run the model. We have updated this repository with a README.md file with instructions on how to setup the code, run the model on a HPC, and process output. This new ReadMe file has been changed in release v1.0.1, and version number has been changed in the manuscript and supplementary information.

The code and data used for the figures can be found in the Zenodo. Zenodo was used instead of GitHub to allow the data and code to be uploaded together, as some of the data files exceeded the file size limits of GitHub. There is already a ReadMe.md file included in the Zenodo, which has a description of the code, instructions on how to run the code, required dependencies, and what code and data is used for each figure.
